# Bilateral External Auditory Exostoses Causing Conductive Hearing Loss: A Case Report and Literature Review of the Surfer’s Ear

**DOI:** 10.7759/cureus.1810

**Published:** 2017-10-30

**Authors:** Dennis A Barbon, Rahul Hegde, Shuo Li, Ahmed Abdelbaki, Divyansh Bajaj

**Affiliations:** 1 Frank H. Netter, Md School of Medicine, Quinnipiac University; 2 Department of Radiology, Yale New Haven Health at Bridgeport Hospital; 3 Diagnostic Radiology, Yale New Haven Health at Bridgeport Hospital; 4 Department of Internal Medicine, St. Vincent Medical Center, Bridgeport, Ct

**Keywords:** external auditory exostoses, surfer's ear, cold water ear, conductive hearing loss, ct, computer tomography, surfer

## Abstract

In patients with repeated exposure to cold water, such as cold water surfers and kayakers, the reactive exostoses can occur in the external auditory canal. The external auditory canal exostoses are multiple, benign bony growths. They can cause external auditory canal stenosis, leading to repeated otitis externa and potentially conductive hearing loss. It is vital to consider this entity in susceptible patients who report hearing loss, as timely intervention such as proper ear protection equipment can lower the risk of developing severe external auditory canal exostoses. We present a case of a 42-year-old male, cold water surfer with conductive hearing loss and bilateral external auditory canal (EAC) stenosis demonstrated on the computed tomography.

## Introduction

External auditory canal (EAC) exostoses are multiple, benign bony growths within the external auditory canal in response to repeated exposure to cold water. They can cause conductive hearing loss and recurrent otitis externa [1]. We present a case of bilateral EAC exostoses in a cold-water surfer, causing conductive hearing loss.

## Case presentation

**Figure 1:**
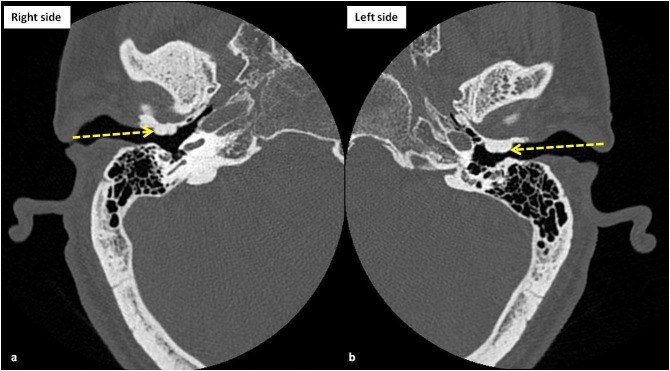
The computerized tomography (CT) scan images (bone windows) of right (a) and left (b) temporal bones at the level of external auditory canals show nearly symmetrical smooth bony outgrowths (yellow arrows), arising from the anterior walls of the external auditory canals, consistent with exostoses. Please note that the exostoses are causing significant narrowing of the external auditory canals leading to the patient’s symptoms of progressive conductive hearing loss, compatible with the diagnosis of surfer's ear.

A 42-year-old male presented to our emergency department (ED) with “hearing problems” as the chief concern. The patient was admitted multiple times for the recurrent ear infections over several months. His ear infections typically began unilaterally, but usually progressed to bilateral involvement. The otorhinolaryngology was consulted in the ED. At this time, Rinne and Weber’s tests showed bone conduction greater than air conduction in both ears, suggesting conductive hearing loss. The patient’s tympanic membranes could not be visualized with an otoscope. The computerized tomography (CT) of the temporal bone was performed which revealed relatively symmetrical bilateral bony outgrowths from the anterior walls of the bilateral external auditory canal (EAC) causing significant canal stenosis, representing bilateral EAC exostoses, consistent with the surfer's ear (Figure 1).

Further investigation revealed that the patient surfed most of his life and has never worn protective ear plugs or neoprene hoods while surfing. Thus, resulting in multiple, bilateral EAC exostosis causing significant external auditory canal stenosis and conductive hearing loss. In concert with the otorhinolaryngology consultation, the final diagnosis was classic surfer’s ear.

## Discussion

The external auditory exostosis (EAE) are multiple, benign bony growth towards the lumen of the external ear canal in response to repeated exposure to cold water. Exostoses form at the suture lines of the tympanic bone, temporal bone, and mastoid bone in the bony canal. Exostoses are commonly found in the patients such as surfers and kayakers, thus earning EAE the moniker of “surfer’s ear”. The external auditory exostosis (EAE) prevalence in cold water surfers ranges between 70% to 80%. Although usually asymptomatic, the complications of the EAE include cerumen impaction, conductive hearing loss, and otitis externa infection [1]. Imaging plays a very important role in the prompt diagnosis of the complications, thus helping in the prompt initiation of the appropriate treatment. However, imaging findings can be nonspecific and it might be difficult to differentiate infections from the malignancies on routine imaging. The diffusion-weighted imaging has been shown to be helpful in differentiating infections from the malignancies by showing central restricted diffusion in the abscesses [2-3]. Once complications arise, the treatment of EAE begins with earplugs and may progress to the canalplasty surgical removal of the exostosis [4]. The surgical treatment can be technically challenging and complications include damage to the tympanic membrane, the facial nerve, and temporomandibular joint. The recurrence of exostoses occurred in eight out of 91 ears that underwent drill canalplasty, with a time-to-recurrence range of one to 15 years [5].

The cold-water theory suggests a causal relationship between the cold water exposure, length of exposure, and the severity of EAE. Possible pathophysiology may be periosteitis following cold water exposure leading to new bone formation. Initially, an anthropological study in 1986 stated that the highest prevalence of EAE occurs in middle latitudes where populations exploit marine and freshwater resources [6]. The current literature, however, supports the cold-water theory. A study of 202 surfers revealed that professional surfers odds ratio (OR 3.8) and predominantly cold water surfers (OR 5.8) were at increased risk of developing EAE. Additionally, the number of years surfed increased the likelihood of developing EAE by 12% annually and increased the risk of developing more severe lesions by 10% annually [1]. A study of 207 United Kingdom surfers found the prevalence and severity of EAE increased significantly after five years of surfing. Interestingly, protective equipment like ear-plugs and hoods statistically decrease the likelihood of severe EAE [7].

It is important to cast a wide differential diagnosis when the patients present with hearing loss. The Rinne and Weber tests are clinical tests used to differentiate between sensorineural and conductive hearing loss. As in our case, the positive Rinne Test indicated the conductive hearing loss in our patient. The list of potential differential diagnoses for conductive hearing loss includes EAE, EAC osteoma, otitis externa, benign polyps, trauma, and the squamous cell carcinoma of the EAC. The EAE and EAC osteoma are both bony lesions of the EAC that may be confused clinically. The EAE was usually medial to the EAC isthmus and bilateral. Unlike EAC osteoma which was lateral, the EAC isthmus was unilateral [8-9].

## Conclusions

In cases related to the bilateral conductive hearing loss, the patient’s occupation, recreational activities, and exposure to cold water should be considered for diagnosis.
